# Metatarsal Head Resections in Diabetic Foot Patients: A Systematic Review

**DOI:** 10.3390/jcm9061845

**Published:** 2020-06-13

**Authors:** Irene Sanz-Corbalán, Aroa Tardáguila-García, Josep M. García-Alamino, Yolanda García-Álvarez, Francisco Javier Álvaro-Afonso, José Luis Lázaro-Martínez

**Affiliations:** 1 Diabetic Foot Unit, Clínica Universitaria de Podología, Facultad de Enfermería, Fisioterapia y Podología, Universidad Complutense de Madrid, 28040 Madrid, Spain; irsanz01@ucm.es (I.S.-C.); yolienf3@hotmail.com (Y.G.-Á.); fraalv@hotmail.com (F.J.Á.-A.); diabetes@ucm.es (J.L.L.-M.); 2 Instituto de Investigación Sanitaria del Hospital Clínico San Carlos (IdISSC), 28040 Madrid, Spain; 3 DPhil Programme in Evidence-Based Healthcare, University of Oxford, Oxford OX1 2JD, UK; josepmariagarciaa@gmail.com

**Keywords:** diabetic foot, metatarsal head resection, reulceration, recurrence, amputation, systematic review

## Abstract

A systematic review and proportional meta-analysis were carried out to investigate the complications that occur after surgical metatarsal head resection in diabetic foot patients. The PRISMA (Preferred Reporting Items for Systematic Reviews and Meta-Analyses) checklist recommendations were applied, and the selected studies were evaluated using a Strengthening the Reporting of Observational studies in Epidemiology (STROBE) checklist. PubMed (Medline) and Embase (Elsevier) were searched in December 2019 to find clinical trials, cohort studies, or case series assessing the efficacy of the metatarsal head resection technique in diabetic foot patients. The systematic review covered 21 studies that satisfied the inclusion criteria and included 483 subjects. The outcomes evaluated were the time to heal, recurrence, reulceration, amputation, and other complications. The proportion of recurrence was 7.2% [confidence interval (CI) 4.0–10.4, *p* < 0.001], that of reulceration was 20.7% (CI 11.6–29.8, *p* < 0.001), and that of amputation was 7.6% (CI 3.4–11.8, *p* < 0.001). A heterogeneity test indicated *I*^2^ = 72.6% (*p* < 0.001) for recurrences, *I*^2^ = 94% (*p* < 0.001) for reulcerations, and *I*^2^ = 79% (*p* < 0.001) for amputations. We conclude that metatarsal head resections in diabetic foot patients are correlated with significant complications, especially reulceration.

## 1. Introduction

Diabetic foot syndrome is a serious condition in Diabetes *mellitus* (DM) that happens to 3–4% of diabetes patients worldwide [1]. The risk of people with DM experiencing a foot ulcer during their lives is 15% [2]. Diabetic foot ulcers (DFUs) located on the plantar surface are one of the main risks in DM [3,4], and those with DFU have a two-times higher five-year mortality risk than those without it [5].

Foot deformities related to diabetic peripheral neuropathy are one of the main causes of DFU underneath the metatarsal plantar heads [6]. Peripheral neuropathy affects the nociceptive reaction, resulting in the development of skin disruption and chronic inflammation [7]. Thus, early detection of the neuropathy using simple screening may help to prevent several complications [8]. Another important risk factor that can lead to DFUs is increased plantar pressure [9].

Unfortunately, ulcer recurrences are common when the ulcer is resolved by surgery. It is estimated that around 40% of patients develop recurrence within one year after the ulcer heals, 60% will develop it within three years, and 65% develop it within five years [10]. Metatarsal head resection (MHR) helps to heal DFUs because it decreases the plantar pressure of the forefoot. Multiple MHR is an effective surgical approach for the management of forefoot deformities, such as rheumatoid arthritis, iatrogenic deformities, and traumatic issue [11].

Hoffman [12] was the first to report this technique for treating claw toes, but he did not discuss the use of this surgery for managing ulceration or osteomyelitis [13].

In 1990, Griffiths and Wieman [14] described MHR for patients with DFU and defined the procedure as a metatarsal head removal by osteotomy at the surgical neck. In addition, they considered that healing was accelerated and recurrence was prevented because of the reduced plantar pressure at the ulcer site [14].

MHR is now a useful surgical process for treating DFU with osteomyelitis in the metatarsal head [14]. The MHR is mainly indicated when X-ray signs are associated with metatarsal bone infection, bone is exposed, rigid deformity is the cause of the ulcer, extensive soft tissue infection is present, and antibiotic therapy and offloading have not shown any result during the past six weeks [15,16]. In the absence of osteomyelitis, MHR is also a viable treatment option for DFUs (e.g., in cases of offloading treatment, hard-to-heal wounds, and bone prominences) [6].

The MHR technique has gradually been modified to obtain better clinical results and fewer postsurgical complications. Despite the procedure, many subjects develop recurrent infection and need an amputation, and an open approach has a higher rate of postsurgical complications [6]. Moreover, this surgical technique modifies the pressures on the next metatarsal head and increases the risk of a new ulcer [17,18]. The consequences of the procedure can be affected by intrinsic technique factors (e.g., the pattern of resection) and intrinsic subjects factors (e.g., underlying diseases) [19]. Therefore, the selection of the surgical procedure (either trans-metatarsal amputation, pan-metatarsal head resection, joint resection, or osteotomy) should be determined by related factors such as patient compliance, vascular status, age, reason for surgery, type of approach, and bone quality [20].

The main objective of several studies has been to evaluate the efficacy of metatarsal resections based on surgical technique rather than complications after MHR. However, the complications related to the procedure may be increased by technique-related factors [19], with the most common complications being pressure transfer lesions, recurrence, infection, and the need for amputation. Therefore, the purpose of the present study is to evaluate the complications after MHR in diabetic foot patients by systematically reviewing the available literature.

## 2. Materials and Methods

A systematic review was carried out using the Preferred Reporting Items for Systematic Reviews and Meta-Analyses (PRISMA) checklist [21] for control. The select studies were evaluated using a Strengthening the Reporting of Observational studies in Epidemiology (STROBE) checklist [22].

### 2.1. Literature Search

PubMed (Medline) and Embase (Elsevier) were searched in December 2019 to find clinical trials, case series, or cohort studies assessing the efficacy of the MHR technique in diabetic foot patients. The keywords used in the search were the following: (‘metatarsal head resection’) AND (‘diabetic foot’). We included studies published in English, Spanish, German, and French. The reference lists of all retrieved studies were cross-checked for additional analysis, and the abstracts of all studies were checked to exclude articles according to our exclusion criteria. Full text reviews were performed to determine whether the remaining studies satisfied the inclusion criteria. The literature review was performed independently by two raters (I.S.-C. and A.T.-G.). Any discrepancies between the two raters were discussed with a third rater (J.L.L.-M.).

### 2.2. Study Selection

We considered clinical trials, case series, or cohort studies assessing patients (>18 years old) with MHR published in English, Spanish, German, and French as inclusion criteria. We excluded in vitro, preclinical or animal studies, trials, non-original articles, reviews, case reports, comments, and letters, as well as studies without available data for analysis. The references of systematic reviews and narratives were examined for additional articles.

### 2.3. Data Collection

Data were extracted using a customised Microsoft Excel spreadsheet. The extracted data included the author, year, study design, presence of a comparative group, number of participants, reason for surgery, surgery technique, surgical approach, time to heal, number of recurrences, number of reulcerations, number of amputations, and other complications.

### 2.4. STROBE Guidelines

The STROBE guidelines were developed to help guarantee high-quality presentation of observational studies [23]. Raters evaluated the items of the STROBE guideline checklist that were adequately reported. This checklist provides a framework to satisfy the need for completeness and transparency. The guidelines have a total of 22 items, of which 18 are common to all three observational designs (cohort, cross-sectional, and case-control). The remaining items (items 6, 12, 14, and 15) have particular variations according to the study design. The following are the STROBE checklist items: item 1–title and abstract; items 2 and 3–introduction; items 4 to 12–methods; items 13 to 17–results; items 18 to 21–discussion; and item 22–funding and sponsorship.

Two raters (I.S.-C. and A.T.-G.) independently assessed each study using the STROBE guidelines. A third rater (J.L.L.-M.) helped to achieve a consensus in cases of disagreement. The raters read the full articles and applied the guidelines. The raters independently familiarised themselves with the STROBE guidelines before starting the reliability test and carried out a joint pilot study. We estimated the degree of agreement between two raters in their appraisals (inter-rater reliability) for all items included in the guidelines.

### 2.5. Statistical Analyses

The proportion of adequately reported items was considered as the number of adequately reported items divided by the total number of applicable items. A proportional meta-analysis was performed using OpenMetaAnalyst software to estimate the overall weighted proportion of each outcome of interest. The data are presented as the overall proportion with a 95% confidence interval (CI) using the random-effects DerSimonian-Laird model. Assessment of the consistency of effects in studies is an essential part of a meta-analysis.

We assessed the heterogeneity using *I*^2^ statistic. The heterogeneity test examines the null hypothesis that all studies are evaluating the same effect. *I* squared (*I*^2^) represents the percentage of total variation in studies due to heterogeneity instead of chance. *I*^2^ can be calculated and compared across meta-analyses with diverse types of studies, sizes, and type of outcomes. Higgins et al. [24] elaborated an approach to quantify the effect of heterogeneity and gave a measure of the degree of inconsistency in the results of these studies.

We calculated the agreement between raters using Fleiss’ kappa (k) statistics for multiple raters. The classification of agreement was as follows [25]: <0: no agreement; between 0 and 0.20: slight; between 0.21 and 0.40: fair; between 0.41 and 0.60: moderate; between 0.61 and 0.80: substantial; and finally, 0.81–1: almost perfect. This statistical analysis was done using various packages in R software.

## 3. Results

### 3.1. Search Results

**Figure 1:**
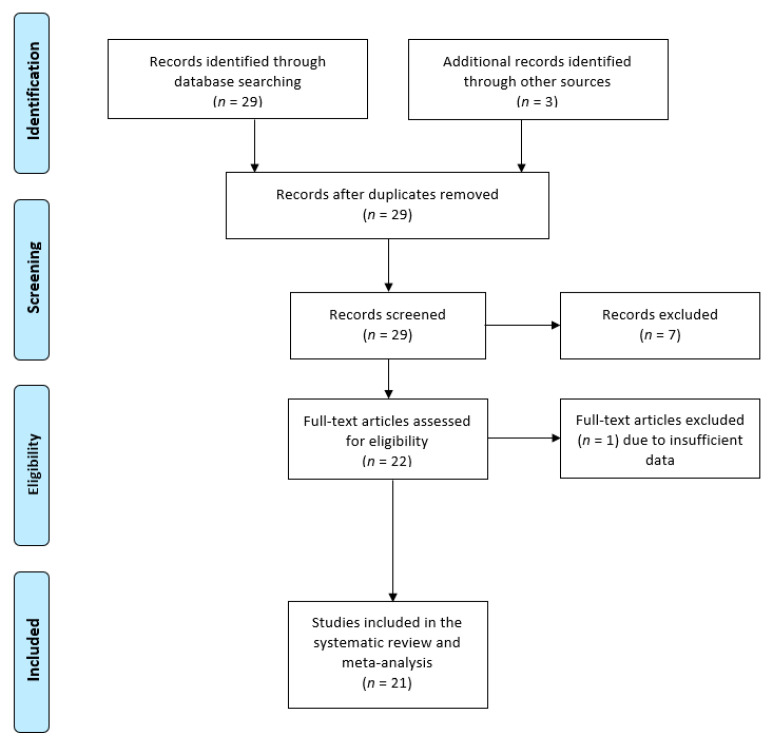
Flow chart of identified studies.

Initially, a total of 29 records were identified in the literature. Titles and abstracts were screened, and 22 studies were potentially eligible for the systematic review. At the end of the screening, 21 studies [3,7,13,14,17,18,19,20,26,27,28,29,30,31,32,33,34,35,36,37,38] met the inclusion criteria and were used in this systematic review (see Figure 1).

### 3.2. Characteristics of the Included Studies

Table 1 summarises the main characteristics of the 21 studies included in the meta-analysis. All of them were published between 1982 and 2019 and were from 6 countries and 13 journals. No randomized controlled trial (RCT) was identified; only case series, observational studies, cohort studies, and case-control studies were found. All of the studies included curative surgery as a surgical indication. Six studies also included the indication for emergency surgery, and only two studies included patients who underwent prophylactic surgery.

The systematic review included a total of 483 patients. The sample of patients included in the studies was small in most. The range of sample size was 2–207 patients, and the mean size was 52.5 ± 50 patients. The patients’ time to heal was only studied in 11 studies, and the mean time was 15.89 ± 6.61 weeks.

**Figure 2:**
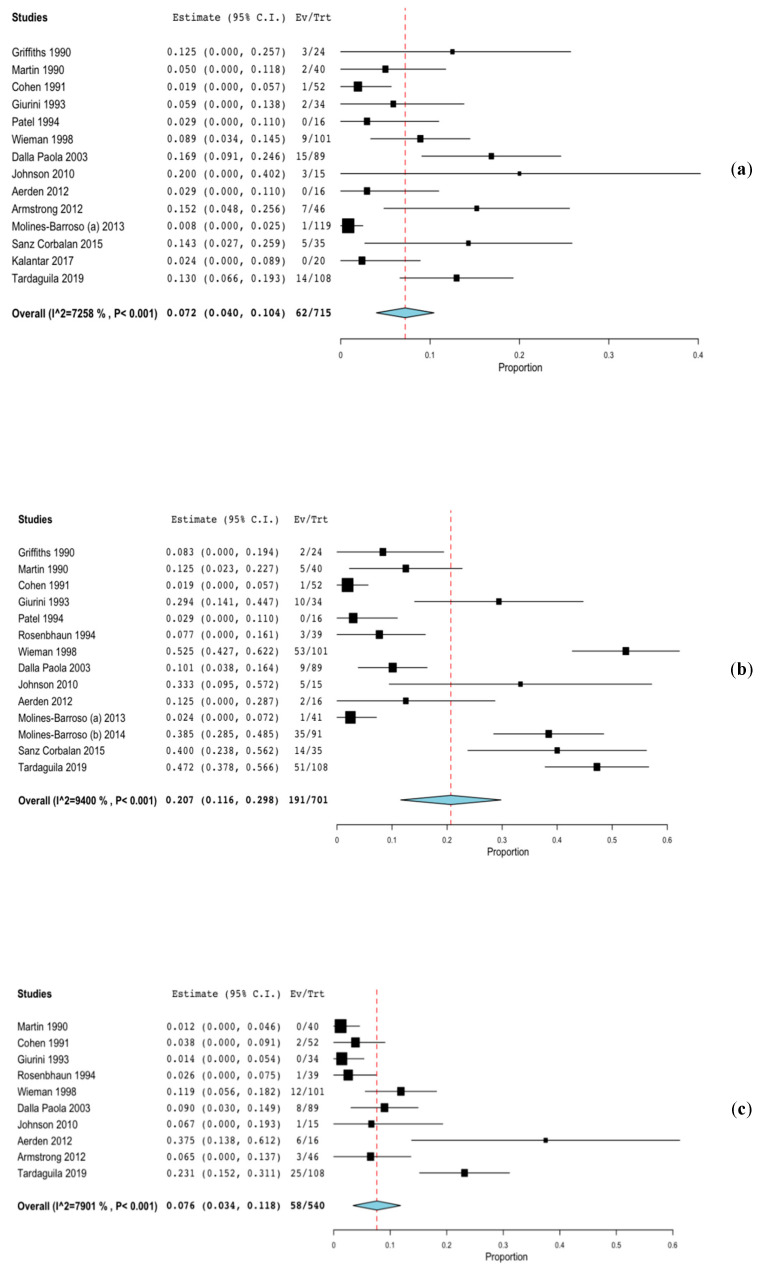
Forest plot—proportion of recurrence, reulceration and amputation. (**a**) Recurrence, (**b**) Reulceration, (**c**) Amputation.

The most frequent surgical approach was the dorsal approach, which was reported in nine studies, followed by the combined plantar and dorsal approach in eight studies, and the plantar approach in only one study. Three studies did not indicate the surgical approach. Other study features are summarised in Table 1. The outcomes evaluated were the time to heal, recurrence, reulceration, amputation, and other complications (see Table 1). The proportion of recurrence was 7.2% (CI 4.0–10.4, *p* < 0.001), that of reulceration was 20.7% (CI 11.6–29.8, *p* < 0.001), and that of amputation was 7.6% (CI 3.4–11.8, *p* < 0.001) (see Figure 2).

### 3.3. Quality of the Reporting

The percentage of STROBE items adequately reported was 51.6% (median 56.5%). Table 2 shows the overall rating for the STROBE checklist. The overall interobserver agreement kappa was good (Fleiss’ Kappa = 0.692).

## 4. Discussion

Our proportional meta-analysis showed that reulceration was present in 20.7% of feet after MHR in diabetic foot patients. However, other complications, such as recurrence and amputation, were only found in approximately 7% of the cases. The literature indicates that reulceration is a frequent event after MHR and should be considered as an intrinsic complication of the surgical intervention [17].

Moreover, after MHR, reulceration appears to be the most prevalent medium- to long-term complication. We found a wide range of rates from 4.5 to 70% [3,14,17,18,26,27,29,30,31,32,33,34,35,38]. Nonetheless, we may consider the time after healing as a remission process rather than one of actually being healed [10]. In addition, a strict podiatric and orthotic follow up will not ensure a reduction of reulceration [32].

Tardáguila-García et al. [3] showed the rate of reulceration ranges from 43 to 53% and depends on the surgical approach. This rate is similar to the rates of 41% seen by Molines-Barroso et al. [17] and 46.7% seen by Sanz-Corbalán et al. [38]. The procedure, follow-up, and characteristics of the patients were similar. On the other hand, the studies with the lowest rate of reulceration were case series without a control group or with a short follow-up [14,28,30,35]. Regarding amputation rates, this surgical technique can be considered as a safe procedure to avoid minor and major amputation in order to salvage limbs.

It is important to note that MHR removes pressure from the metatarsal head but also transfers this pressure to contiguous metatarsal heads, resulting in reulceration [17]. However, only two studies [19,20] compared MHR with conservative treatment, and both of them showed lower numbers of patients with reulceration in the surgical group. Recurrence was evaluated in different studies, but only one of them analysed why the recurrence appears after MHR [38]. According to the conclusion of that paper [38], MHR of less than 25% of the length of the metatarsal is associated with recurrence after surgery. This conclusion could explain the low proportion of recurrences. Early recurrences are probably related to residual osteomyelitis, so long-term follow-up is important to register recurrences properly (12 months is enough time to consider the osteomyelitis as healed [39]). After MHR, recurrence occurs in the range of 0–17% [3,7,14,17,19,26,27,28,29,30,32,33,34,35,38].

With regard to amputation, the records in different studies are variable and limited. We think that the follow-up time was not extensive in general. Two papers showed the highest amputation rates [3,32]. The main difference between the papers is that Wieman et al. [32] identified 12 (11.9%) amputations, which were all major, and Tardáguila-García et al. [3] identified 25 (23.1%) amputations, but only 3 (2.8%) were major. The differences in the percentage of major amputations could be related to the type of surgery (e.g., pan-resection or first MHR). To our knowledge, this is the first systematic review and proportional meta-analysis to estimate complications after MHR in diabetic foot patients. Therefore, it is not possible to make comparisons with other similar studies.

### 4.1. Importance of the Study: Possible Mechanisms and Implications for Clinicians or Policy Markers

The transfer of these results to clinical applications should involve the consideration of reulceration as an implicit complication of MHR. This study could help to raise awareness among clinicians that the rates of reulceration can be high in patients with MHR and that the main objective of studies related to these surgeries should be to reduce reulceration in these patients. This concern must be taken into account when performing an MHR in non-specialised areas or when a patient shows previous amputations and rigid joint deformities because the number of reulcerations may increase considerably over time. For this reason, very important factors to take into account to reduce reulceration rates after MHR are an intensive follow-up programme, the design of individualised orthopaedic treatments by a podiatrist specialised in diabetic feet, and evaluation of the proportion of previous amputations and structured joint deformities. A strict podiatric and orthotic follow-up may not reduce reulceration [32], so it is important to consider the patient to be in remission instead of healed [10].

Nevertheless, MHR has several advantages. For instance, it can be performed with local anaesthesia, which has less risk. Furthermore, it is a single procedure, and the wound could be closed by suture. It could lead to a quick return to weight bearing with a short disability period and easy recovery, and it could be a good option for treating patients with ulcers complicated by osteomyelitis [40].

### 4.2. Strengths of the Study

The main strength of this study is the in-depth review of the literature, which contributes to the more accurate estimation of the prevalence than other studies that only show averages and percentages as results. The proportional meta-analysis also allows for the estimation of heterogeneity between different studies.

### 4.3. Weaknesses of the Study

The first limitation is the lack of some information in the studies, which is also shown in other meta-analyses and makes it difficult to evaluate some variables. For instance, it was impossible to perform a sub-group analysis comparing dorsal and plantar approaches, different types of surgery, or the presence or absence of infection. Having this data could increase the information provided by the review.

The second limitation was the lack of a randomised control with two or more comparison groups to evaluate this surgical technique.

The third limitation is related to the data extraction because three of the authors of the present study were involved in some of the individual studies included. However, we consider that the impact of this point is low due to the definition of the outcome and type of data.

The fourth limitation was high statistical heterogeneity. The heterogeneity test indicated *I*^2^ = 72.6% (*p* < 0.001) for recurrences, *I*^2^ = 94% (*p* < 0.001) for reulcerations, and *I*^2^ = 79% (*p* < 0.001) for amputations. From the information available, it is difficult to determine an objective cause that would explain this heterogeneity. However, it could be related to the follow-up time after MHR because complications do not usually develop immediately, and it is necessary to perform medium or long-term follow-up to observe several complications. In addition, the different methodologies presented in the publications also complicate the homogenisation of results. It was not possible to perform a sensitivity analysis, and the available data did not allow for a meta-regression analysis to be performed.

### 4.4. Unanswered Questions And Future Research

More controlled high-quality studies are required of several characteristics of ulcers with regard to the type of surgery, MHR approach, surgical technique, the presence of infection, evolution of the ulcer, local treatment received, offloading received, and other parameters that may contribute to explaining the differences in the development of complications.

## 5. Conclusions

Based on the available evidence, we conclude that MHR is a popular and useful surgical technique for treating diabetic foot-related problems. However, we have to consider that MHR has significant complications, especially reulceration. More control is needed when performing medium or long-term follow-up in order to record complications due to MHR properly.

## Figures and Tables

**Table 1:** Characteristics of studies included in the systematic review.

Author/Year	Study Design	Comparative Group	Number of Participants	Reason of Surgery	Surgery Technique	Surgical Approach	Time until Healing	Number of Recurrences	Number of Reulcerations	Number of Amputations	Evaluated other Complications
Jacobs RL/1982	Case series	No	12	Curative and emergent	Clayton modification (Hoffman)	Dorsal	-	-	-	-	-
Giurini JM/1987	Case series	No	15	Curative	Pan-metatarsal head resection	Dorsal	-	1	-	-	Yes
Griffiths GD/1990	Retrospective	No	24	Curative and prophylactic	MHR	Dorsal	10.4 ± 6.9 weeks	3	2	-	No
Martin JD/1990	Prospective observational	No	40	Curative	MHR	Dorsal	3 weeks	2	5	0	No
Cohen M/1991	Retrospective	No	52	Curative and urgent	Partial ray resection. Trans-metatarsal amputation. Pan-metatarsal head resection.	Dorsal and plantar	-	1	1	2	Yes
Giurini JM/1993	Retrospective	No	34	Curative	Pan-metatarsal head resection	Dorsal and plantar	-	2	10	0	Yes
Patel VG/1994	Before and after	No	16	Curative	MHR	-	8.0± 2.0 weeks	0	0	-	No
Rosenblum BI/1994	Retrospective	No	39	Curative and urgent	MHR. Metatarsal osteotomy. Pan-metatarsal head resection.	Dorsal and plantar	92 (8.6–278.1) weeks	-	3	1	Yes
Wieman TJ/1998	Prospective observational	No	101	Curative and prophylactic	MHR	Dorsal	12 weeks	9	53	12	Yes
Dalla Paola L/2003	Cohort	No	89	Curative and emergent	First ray amputation	-	-	15	9	8	No
Armstrong DG/2005	Cohort	Yes	22	Curative	Fifth metatarsal head resection	Dorsal	5.8 ± 2.9 weeks	-	-	-	Yes
Johnson JE/2010	Retrospective	No	15	Curative	First meta-tarso-phalangeal joint resection and pin stabilization	Dorsal and plantar	-	3	5	1	No
Aerden D/2012	Prospective observational	No	16	Curative	First metatarsal head resection	Dorsal and plantar	6.2 (1.2–9.8) weeks	0	2	6	Yes
Armstrong DG/2012	Control and cases	Yes	46	Curative	Pan-metatarsal head resection	Dorsal	12.0 ± 5.7 weeks	7	-	3	Yes
Faglia E/2012	Retrospective	Yes	207	Curative	MHR. Ray amputation.	Dorsal and plantar	-	-	-	-	Yes
Molines-Barroso RJ/2013	Prospective observational	No	119	Curative and emergent	MHR	-	-	1	41	-	No
Boffeli TJ/2014	Case series	No	2	Curative	Pan-metatarsal head resection	Plantar	6 weeks	-	-	-	Yes
Molines-Barroso RJ/2014	Prospective observational	Yes	91	Curative and emergent	MHR	Dorsal and plantar	-	-	35	-	No
Sanz-Corbalán I/2015	Prospective observational	No	35	Curative	MHR	Dorsal	-	5	14	-	No
Kalantar Motamedi A/2017	Cohort	Yes	20	Curative	MHR	Dorsal	5.3 ± 4.6 weeks	0	-	-	Yes
Tardáguila-García A/2019	Retrospective	Yes	108	Curative	MHR	Dorsal and plantar	14.1 ± 10.2 weeks	14	51	25	Yes

Abbreviation: MHR, metatarsal head resection.

**Table 2:** Overall rating for Strengthening the Reporting of Observational studies in Epidemiology (STROBE) checklist.

	Item Number–STROBE Guidelines																						
	1		2	3	4	5	6	7	8	9	10	11	12	13	14	15	16	17	18	19	20	21	22
	Title	Abstract																					
Jacobs RL/1982	Yes	Yes	Yes	No	No	No	No	No	No	No	No	No	No	Yes	Yes	No	No	No	No	Yes	Yes	No	No
Giurini JM/1987	No	Yes	Yes	No	No	No	No	No	No	No	No	Yes	No	No	Yes	Yes	No	No	Yes	No	Yes	No	No
Griffiths GD/1990	No	No	Yes	Yes	Yes	Yes	No	No	No	No	No	Yes	No	Yes	Yes	Yes	No	No	Yes	No	Yes	No	No
Martin JD/1990	No	Yes	Yes	No	No	No	No	No	No	No	No	No	No	Yes	Yes	Yes	No	No	Yes	No	No	No	No
Cohen M/1991	Yes	No	Yes	No	Yes	Yes	No	No	No	No	No	Yes	No	Yes	Yes	Yes	No	No	Yes	No	No	No	No
Giurini JM/1993	Yes	Yes	Yes	No	Yes	Yes	Yes	Yes	No	No	No	Yes	No	Yes	Yes	Yes	No	No	No	No	Yes	No	No
Patel VG/1994	No	Yes	Yes	Yes	No	No	No	Yes	Yes	Yes	No	Yes	Yes	Yes	Yes	Yes	Yes	Yes	Yes	No	Yes	No	No
Rosenblum BI/1994	No	Yes	Yes	No	No	Yes	No	Yes	Yes	No	No	Yes	No	Yes	Yes	Yes	No	No	No	Yes	Yes	No	No
Wieman TJ/1998	Yes	Yes	Yes	Yes	No	Yes	No	Yes	Yes	No	No	Yes	Yes	Yes	Yes	Yes	Yes	Yes	Yes	Yes	Yes	No	No
Dalla Paola L/2003	Yes	Yes	Yes	Yes	No	Yes	Yes	Yes	Yes	Yes	No	Yes	No	Yes	Yes	Yes	No	No	Yes	No	Yes	No	No
Armstrong DG/2005	Yes	Yes	Yes	Yes	Yes	Yes	Yes	No	No	No	No	Yes	Yes	No	Yes	Yes	Yes	No	Yes	Yes	Yes	No	Yes
Johnson JE/2010	Yes	Yes	Yes	Yes	Yes	Yes	Yes	Yes	Yes	No	No	No	No	Yes	Yes	Yes	No	No	Yes	Yes	Yes	No	No
Aerden D/2012	Yes	Yes	Yes	No	Yes	Yes	Yes	Yes	No	No	No	Yes	No	Yes	Yes	Yes	No	No	Yes	Yes	Yes	No	Yes
Armstrong DG/2012	No	Yes	Yes	Yes	Yes	No	Yes	Yes	Yes	No	No	Yes	Yes	Yes	Yes	Yes	Yes	Yes	Yes	Yes	Yes	No	No
Faglia E/2012	No	Yes	Yes	Yes	No	Yes	No	Yes	Yes	No	No	Yes	Yes	Yes	Yes	Yes	No	Yes	Yes	No	Yes	No	Yes
Molines-Barroso RJ/2013	No	Yes	Yes	Yes	Yes	No	Yes	Yes	Yes	No	No	Yes	Yes	Yes	Yes	Yes	No	No	Yes	Yes	Yes	No	Yes
Boffeli TJ/2014	Yes	Yes	Yes	Yes	Yes	No	No	Yes	No	No	No	No	No	Yes	Yes	Yes	No	No	Yes	No	Yes	No	Yes
Molines-Barroso RJ/2014	No	Yes	Yes	Yes	Yes	Yes	Yes	Yes	Yes	No	No	Yes	Yes	Yes	Yes	Yes	Yes	Yes	Yes	Yes	Yes	Yes	Yes
Sanz-Corbalán I/2015	Yes	Yes	Yes	Yes	Yes	Yes	Yes	Yes	Yes	No	No	Yes	Yes	Yes	Yes	Yes	Yes	Yes	Yes	Yes	Yes	No	Yes
Kalantar Motamedi A/2017	Yes	Yes	Yes	Yes	Yes	Yes	Yes	Yes	Yes	No	No	Yes	Yes	Yes	Yes	Yes	Yes	Yes	Yes	Yes	Yes	No	Yes
Tardáguila-García A/2019	Yes	Yes	Yes	Yes	Yes	Yes	Yes	Yes	Yes	No	No	Yes	Yes	Yes	Yes	Yes	Yes	Yes	Yes	Yes	Yes	No	No
